# Medicinal Plants for Management of Insomnia: A Systematic Review of Animal and Human Studies

**DOI:** 10.31661/gmj.v8i0.1085

**Published:** 2019-01-01

**Authors:** Faezeh Feizi, Nazli Namazi, Roja Rahimi, Mohammad Hossein Ayati

**Affiliations:** ^1^School of Pharmacy, Islamic Azad Medical University, Tehran, Iran; ^2^Obesity and Eating Habits Research Center, Endocrinology and Metabolism Molecular- Cellular Sciences Institute, Tehran University of Medical Sciences, Tehran, Iran; ^3^Department of Traditional Pharmacy, School of Traditional Medicine, Tehran University of Medical Sciences, Tehran, Iran; ^4^Department of Traditional Medicine, School of Traditional Medicine, Tehran University of Medical Sciences, Tehran, Iran

**Keywords:** Insomnia, Sleep, Herbs, Plants

## Abstract

Insomnia is one of the most troubling sleep disorders and can be characterized by an inability to fall asleep and/or inadequate sleep duration and/or waking up multiple times during the night. Herbal medicine has been used to treat a range of sleep disorders for centuries. This study aimed to review medicinal plants investigated experimentally or clinically for sleep disorders, as well as their potential mechanisms of action and active components. Electronic databases and literature were systematically investigated to assess all in vitro and in vivo trials and clinical evidence of the efficacy and potential mechanisms of actions playing major roles in sleep induction or insomnia treatment. Among many herbal studies and trials on insomnia, some showed no significant difference between herbal remedies and placebos. While others showed improvements in sleep parameters (sleep latency, total sleep, non-rapid eye movement (NREM) and rapid eye movement (REM) sleep duration, delta activity in NREM sleep, wakefulness anxiety-associated insomnia). In this study, in vitro, animal, and clinical studies investigating a variety of herbal treatments for insomnia were systematically reviewed. The mechanisms of action of herbal medicines in treating insomnia are mainly related to gamma-aminobutyric acid (GABA)-synthesizing and GABA-metabolizing enzymes that influenced sleep outcomes. Overall, herbal remedies were not associated with more benefits than nonbenzodiazepines, although side effects were less. The results suggest that herbs have some benefits in improving the quantity and quality of sleep and could be a promising alternative therapy.

## Introduction

Insomnia is one of the most troubling sleep disorders and can be characterized by an inability to fall asleep and/or inadequate sleep duration and/or waking up multiple times during the night. It can be caused by several factors leading to circadian rhythm disturbances. This issue may be a significant contributor to the progression of several neurological or non-neurological disorders. Recently, it has been reported that insomnia may play an influential role in the incidence of Alzheimer disease, suicidal ideation, anxiety, obesity, hypertension, and diabetes mellitus [1]. Insomnia can be caused by different neurological disorders, such as Parkinson disease, restless legs syndrome, depression, gastrointestinal problems, and several endocrine disorders. Insomnia can be managed using medication appropriate for the main cause of insomnia [2]. There have been various chemical medicinal interventions so far, with proven efficacy in rectifying patients’ sleep patterns. Benzodiazepine (BZD) receptor agonists, melatonin and orexin receptor antagonists, and histamine antagonists (selective H1 antagonists such as doxepin) are the most commonly indicated drugs for treating insomnia. Orexin receptor antagonists may cause somnolence, fatigue, and dry mouth in patients. Ramelteon (melatonin receptor agonist) likely promotes sleep by acting on the melatonin receptor 1A (MT1) by attenuating wake-promoting signals from the suprachiasmatic nucleus and might influence the timing of sleep via the melatonin receptor 1B (MT2). The off-label use of antidepressants may be proven effective, even though various adverse effects such as suicidal ideation may occur in patients. Mirtazapine (which promotes sleep via antagonism of serotonin 5HT2 and 5HT3, histamine H1, and alpha-1-adrenergic receptor antagonists), and alpha-1-adrenergic antagonists such as prazosin can also be indicated (off-label use), being an antihypertensive agent used in the treatment of nightmares and sleep disturbances in patients with posttraumatic stress disorder (PTSD). Antipsychotics can also be used for treating insomnia (off-label use) but may cause sedation, blurred vision, dizziness, dry mouth, and urinary inconsistencies in the short term and increased appetite and subsequent weight gain in the long-term. Anticonvulsant drugs used to treat insomnia include gabapentin, pregabalin, and, less frequently, tiagabine [3]. The major caveat of these interventions is their associated adverse effects and consequently low patient compliance, causing maltreated insomnia. To date, the routine drugs for insomnia cause drowsiness during the daytime and disturbances in activities that require cognition and consciousness. Moreover, a majority of patients become tolerant to certain drugs, which leads to significantly higher consumption of sleeping pills, along with anxiolytic agents such as alprazolam [4]. Thereby, additional approaches are needed to treat or manage sleep disorders. The use of natural products, including herbs, is increasing across the world. International organizations, such as the World Health Organization (WHO), are also making more efforts and paying more attention to the development and promotion of the quality of natural products [5]. Additionally, non-pharmaceutical therapies, especially for mild to moderate conditions and symptoms such as non-severe insomnia and for the elderly, are highly recommended as first-line therapies prior to chemical medication [6]. Owing to their cost-effectiveness, easy access, and lower side effects, medicinal plants are popular across the world. Medicinal herbs, regardless of their probable impurities, are believed to contain effective agents for the treatment of insomnia and other sleep disorders [7]. This study aimed to review medicinal plants investigated experimentally or clinically for sleep disorders, as well as their potential mechanisms of action and active components.

## Search Strategies

**Figure 1:**
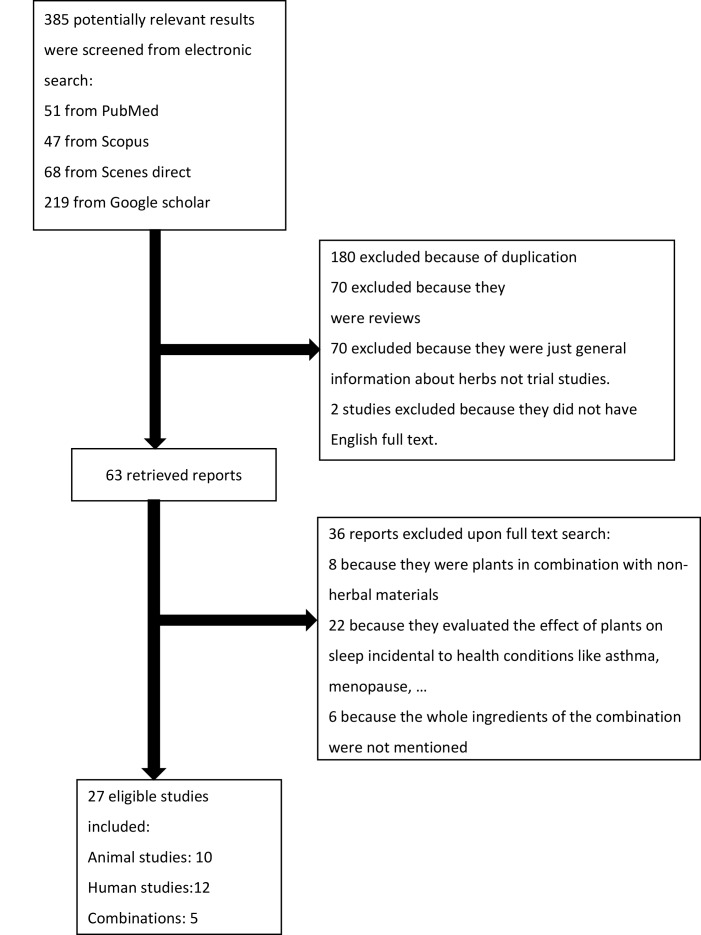
Study Selection Diagram

Electronic databases including PubMed, Scopus, Web of Science, and Google Scholar, were searched over a period from 1990 to 2016. All studies retrieved were investigated to assess all reported in vitro and in vivo trials or clinical evidence of the efficacy and potential mechanisms of action which played a major role in the induction of sleep or treatment of insomnia. In this review, only published reports and literature in English were included. The search terms were “insomnia” or “sleep” or “plant” or “herb” or “extract” in the title. Articles in languages other than English, review articles, studies on the mixture of plants and other agents, experimental studies on plants without relevant biological effects, case reports, and case-control studies were excluded. Articles without full texts were also excluded. Figure-1 shows the study selection process. The reference lists of the final selected reports were also reviewed to find other pertinent studies. The selected articles were studied to retrieve the plant’s scientific name, part used, type of extract used, active components, and type of animal model (for in vivo studies). The authors searched for alterations in test groups compared with control groups in sleep related-parameters such as sleep latency, sleep maintenance, total sleep duration, wakefulness, and effects on sleep waves. In the studies on humans, study design, Jadad score, interventions, duration of treatment, and data related to the efficacy and tolerability of the patients on the herbal treatment were also collected. The ↑ and ↓ signs were used to indicate significant increases and decreases in the implied variables, respectively. From a total of 385 results, 180 studies were excluded because of duplication (from different databases). Seventy studies were excluded because they were review articles. Seventy studies were excluded because they were general information about herbs, not trial studies. Two studies were excluded because they did not have English full text. Thirty-six studies were excluded based on their title and abstract: of these, eight were related to the use of plants in combination with non-herbal materials, 22 evaluated the effects of plants on sleep disorders incidental to health conditions, such as asthma and menopause, and six did not mention the complete ingredients of the combination. Figure-1 shows the diagram of the study selection process.

## Results

In Tables 1-3, the medicinal herbs mentioned for the management of insomnia and all pieces of evidence confirming their efficacy are described individually. According to the latest literature on medicinal plants and their applications in treating insomnia, in both humans and animals, the following plants and their related information can be found in Table-1. *Matricaria recutita* had modest benefits on daytime function, low sleep latency, and nighttime awakening. The possible mechanism could be due to the effects of the flavonoid component. It has been demonstrated that it can modulate gamma-aminobutyric acid (GABA) receptors [8]. It has been shown that *Melissa officinalis* (lemon balm) caused significant improvements in insomnia: anxiety manifestations and anxiety-associated symptoms. The components of lemon balm, including rosmarinic acid, pentacyclic triterpenoids, ursolic and oleanolic acids, might act as inhibitors of GABA catabolism [9]. Moreover, in a clinical trial on *Piper methysticum* (kava), non-psychotic anxiety sleep disorders can be treated efficiently and safely with kava extract (WSR 1490) [9].Other clinical trials did not indicate positive effects of medicinal herbs on insomnia. Two clinical trials on *Lavandulaangustifolia* (lavender) [10, 11] did not show significant beneficial effects. Lillehei *et al*. found that lavender made the patients feel refreshed after waking up. However, it had no useful effect on sleep quantity [10]. Additionally, Lewith *et al*. concluded that lavender improved sleep quality in women and younger subjects. However, its effect was not significant [11]. According to the study by Ngan *et al*., *Passiflora* had only short-term benefits on sleep quality.*Passiflora incarnata* (passionflower) also showed no significant changes in the polysomnography (PSG) and anxiety parameters [13]. Although *Xylaria nigripes* indicated no significant differences compared with a placebo, an intracomparison revealed an improvement in the intervention group. The researchers suggested that the active component, 5-methylmellein, can increase GABA in the brain and improve insomnia [14]. Three studies evaluated the effects of*Valeriana officinalis* (valerian) in human models. Coxeter *et al*. reported that valerian lowered the falling asleep time and had a minor effect on sleep quality. In another study, it was shown that this plant caused long-term slow-wave sleep (important in recovery) in non-rapid eye movement (NREM) sleep in the intervention group, compared with a placebo. It also showed positive effects on mild psychological insomnia [17]. However, in another study, no significant difference was shown between 300 mg/day and 600 mg/day valerian tablets and placebos regarding recorded sleep electroencephalography (EEG), mood, and psychometric performance [18]. Another medicinal herb is*Valeriana edulis*(*Valeriana mexicana*). It has been indicated that *V. mexicana* increased rapid eye movement (REM) sleep (more than *V. officinalis*) and delta activity, while it decreased the duration of stage 1 and 2 in NREM sleep, sleep latency, awakening episodes, and morning drowsiness (more than *V. officinalis*) [19].

### Animal Studies

Eleven animal studies were included in the current systematic review (Table-2). *P. methysticum* increased delta activity in NREM sleep (against flunitrazepam) in rats. However, it had no effect on the total waking and NREM sleep time. It also significantly decreased sleep latency. The extract was not mediated by the BZD receptors (because the effect was not antagonized with BZD antidotes such as flumazenil) [20]. An experimental study showed that *Matricaria chamomilla* or *P. incarnata* decreased the sleep latency and then antagonized by 3mg/kg flumazenil (a BZD antagonist); thus, it is possible to calculate thatchamomilehas BZD-like hypnotic activity. However, it had positive effects on neither NREM nor REM sleep. Also, delta activity and wakefulness time did not change considerably after the intervention [21]. *V. officinalis* decreased sleep latency (dose-dependent) and delta activity in NREM sleep. However, it had no effect on NREM, REM, and wakefulness [22]. *Coriandrum sativum*, hydro-alcoholic extract (HAE) and ethyl acetate fraction (EAF), N-butanol fraction (NBF) (HAE and its three fractions, water fraction [WF], EAF, and NBF were prepared from *C. sativum*) increased sleep duration, and NBF decreased sleep latency. NBF showed the highest hypnotic activity and no neurotoxic effect [25]. It has been demonstrated that *Hypericum perforatum* (St. John’s wort) increased body weight, locomotor activity, and antianxiety effect, but it decreased oxidation damage. When co-administered with imipramine, greater improvement was seen. The compounds in this plant inhibit serotonin dopamine norepinephrine reuptake (similar to Selective Serotonin Reuptake Inhibitors [SSRIs] and Tricyclic Antidepressants [TCAs]) and have anti-oxidative effects through polyphenolic acids and flavonoids. Imipramine, as well as St. John’s wort, has antianxiety effects [24]. *Panax ginseng* (Korean red ginseng) increased total sleep and NREM sleep and decreased wakefulness and sleep-wake cycles. However, α-wave activity increased during NREM and REM sleep. Additionally, the expression of α- and β-subunits of GABA receptors decreased. The plausible mechanism is its impact upon the GABAergic systems [26]. A dose of 10 mg/kg of *P. ginseng* (Korean red ginseng) enhanced α-wave activity and lowered δ-wave activity in REM and NREM sleep. It also significantly decreased NREM and total sleep. A dose of 50 mg/kg acted like a 10 mg/kg dose, without any notable effect on REM sleep. It is worth noting that 100 mg/kg dosage indicated no effect on EEG waves; it only increased total sleep. Lower doses of red ginseng extract (RGE) were more effective for sleep regulation, specifically on NREM sleep [27]. Valerian preparation (BIM) decreased sleep latency significantly (1000 mg of each, with greater effect from valerian than BIM). However, it had no effect on NREM, REM, wakefulness and delta activity in NREM sleep for both. It increased the GABA level [28]. *Stephania tetrandra* (40 mg/kg, 80 mg/kg) decreased sleep latency in NREM and increased the amount and duration of NREM sleep. It increased wakefulness. The increment of the dosage from 40 to 80 mg/kg resulted in an increase of the number of state alterations from wakefulness to NREM sleep and from NREM sleep to wakefulness. It may have mixed partial dopamine D1 receptor agonist/full D2 antagonist properties. Moreover, it stimulated NREM sleep. The blockade of D2R plays a key role in the hypnotic action of stepholidine (the active compound in *S. tetrandra*) and initiation of sleep-active neurons in the ventrolateral preoptic nucleus VLPO [29]. There have been combination therapies studied with several factors, which are mentioned in Table-3.

## Discussion

Several studies are indicating that medicinal plants had a significant effect in treating insomnia, and some studies failed to report a significant efficacy. Medicinal plants might have a therapeutic effect on the circadian rhythm, the quality of sleep, sleep maintenance, and the quality of wakefulness and alertness after waking up. Hence, due to their fewer side effects and reasonable efficacy, they may be good choices for the treatment of insomnia, in single usage or when used as a combination with approved medications. The disagreement for their efficacy indicates a requirement for further investigations to shed light on the underlying mechanisms of medicinal herbs in treating insomnia. Additionally, the synergic effects of active compounds, co-administration of different herbal medicines, and application route should be subjects of future investigations.

### Limitations and Strengths

Our systematic review had some limitations. Insufficient number of the studies did not allow us to explore the effects of disease background and gender on the efficacy of herbs on insomnia. In most of the trials, lifestyle was not considered, and no sufficient details about diet were mentioned. These issues may have affected the overall effect sizes. Besides, the review did not cover resources such as books, dissertations, and government reports and non-English publications. Future studies should consider publications with no language limitation and cover gray literature apart from easily accessible international databases. The strength of the current systematic review was to summarize the effects of medicinal herbs on insomnia management, derived from both animal and human studies.

## Conclusion

Of the ten clinical trials, seven had high quality methodology (score>3). However, due to the limited studies for each medicinal herb, it is not possible to draw a fixed conclusion about the efficacy of herbs on insomnia. Further clinical trials are needed to clarify the positive effects of each medicinal plant on sleep quality and quantity. The discrepancy in studies which evaluated the same herb might be due to differences in study participants, study design, and dosage and duration of the intervention. Concurrently, it is critical to establish the safety and efficacy of herbal medicines for treating insomnia in short- and long-term studies, for the wider application of herbal medicines for sleep disorders. Without these studies, they may potentially result in ineffective agents being used for treating insomnia. Therefore, unless serious mechanistic studies are undertaken to elucidate the mechanisms of action, there will be hesitation regarding the usage of medicinal herbs for treating neurological disorders such as insomnia.

## Acknowledgment

The authors express their special thanks to Dr. Mahdi Rezayat, Dr. Sepideh Arbabi Bidgoli, and Dr. Gholamreza Amin for their generous comments and support.

## Conflict of Interest

None declared.

**Table 1:** Clinical Trials on the Efficacy and Mechanism of Action of Certain Medicinal Plants for Treatment of Insomnia

**Scientific name (common name)**	**Part (extract)**	**Study design**	**Jadud** **score**	**Intervention**	**Patients**	**Duration**	**Result**	**Mechanism**	**Reference**
**Matricaria recutita** **(German chamomile)**	Flowering tops (90 mg chamomile extract:3.9 mg of apigenin and 1.8 mg of (-a-bisabolol)	Randomized, double-blind, placebo-controlled pilot trial	5	270 mg twice daily(n=17)/ received placebo(n=17)	34 patients aged 18-65 years with DSM-IV primary insomnia for ≥ 6-months	28days	Modest benefits of daytime functioning and ↓sleep latency and nighttime awakenings	Flavonoid constituent apigenin sedate by modulation of GABA receptors	8
**Piper methysticum** **(kava)**	WS®1490: 100mg dried root of kava 70% kava(acetonic water) and ancillary30% to up absorption	Randomized, double-blind, placebo-controlled trial	4	Received 200 mg daily in the evening(n=34) / placebo(n=27)	61 patients suffered from various anxiety and insomnia on criteria (DSM-III-R)	1week singles blind +4weeks double-blind treatment	Non-psychotic anxiety sleep disorders can be effectively and Safely treated with kava extract WSR 1490®	no mechanism of action presented	9
**Lavandula angustifolia** **(English lavender)**		Randomized, double-blind controlled trial	5	Received good sleep hygiene and an inhalation patch with 55 µl of lavender on their chest(n=39) Control group: received good sleep hygiene and blank patch(n=40	79 college students with self-reported sleep issues	5nightsand 2weeks following up	Waking feeling refresher, no difference in sleep quantity	no mechanism of action presented	10
**L. augustifolia**	Flower	Randomized, Single-Blinded trial	5	6–8 drops of each oil were added each night to the cartridge -100% hydrosoil lavender(n=5) -almond oil for control(n=5)	5male 5female aged 18-50y. with mild insomnia evaluated by (PSQI)with a global score of >5	4weeks: 2w is watched out between per 1weeks as treatment	Better improvement for Women and younger volunteers	no mechanism of action presented	11
**Melissa officinalis** **(lemon balm)**	Cyracos®: Hydroalcholic Leaf extract 7% rosmarinic acid and greater than 15% hydroxycinnamic acid derivatives	a prospective, open-label	0	300 mg cyracos twice in morning and evening	20 stressed volunteers based on DSM-IV-TR (18- 70) years	5days	Significant improve- ment in all categories studied: anxiety manifestations, anxiety-associated symptoms and insomnia	Rosmarinus acid and the pentacyclic triterpenoids, ursolic and oleanolic acids which inhibit GABA catabolism	12
**Passiflora** **Incarnata** **(passionflower)**	leaves, stems, seeds and flowers	Double-blind, Placebo-controlled Repeated measures	2	-Passionflower Tea bags: 2 g of dried plant equivalent to 250 mL by boiled water -Placebo: parsley teabags (each containing 2 g of dried Petroselinum crispum)	41 volunteers (18-35y)with extreme sleep difficulties	22 days study with 7 days washout period (8-14)	Has short-term benefits in sleep quality -no significant changes in any of the PSG parameters and anxiety parameters	no mechanism of action presented	13
**Xylaria nigripes**	Wuling ®capsule: mycelium	Randomized, double-blind, placebo-controlled	5	To take Wuling capsule (0.33 g per-capsule and 63 capsules in one bottle) or identical placebo capsule (3 capsules orally, 3 times daily)	212 cases of insomniac Patients (ICD-10) 18-60Y	4weeks treatment and 2 weeks following up	No significant difference between placebo and Wuling Effective in compare to pre and post treatment	↑ intake of GABA in brain active compound: 5-methylmellein	14
**Valeriana officinalis** **(valerian)**	Valerina Forte®: 200mg dry root and rhizome	Televised, Web-Based Randomized, double-blind trial	5	In per group of treatment(n=202) and control(n=203), Patients received a box with 60 tablets to swallow 3 tablets one hour before going to bed	405 participant(18-75y)had insomnia Based on (PSQI) score of >5	2weeks	-↓ fall asleep time -small effect on sleep quality	no mechanism of action presented	15
**V. officinalis**	Tablets contained 225mg V. Officinalis root and rhizome extract (2.94mg total Valerenic acids,0.46mg Valerenal and 1.23mg Valtrates)	Randomized, double-blind, placebo-controlled, crossover trial	5	2tab(225mg valerian) half an hour before bed	42 enrolled patient (22-75)	6weeks	No better than placebo in promoting sleep-related factors	no mechanism of action presented	16
**V. officinalis**	Sedonium®: 300 mg dry root of radix valerianae	Randomized, double-blind, placebo-controlled, crossover trial	2	2tab one hour before bed	16 patients (22-55Y) with psychophysiological insomnia (ICSD-CODE 1.A.1.)	8 study nights were scheduled in two trial periods, separated by a washout period of 12 days	-In long-term slow wave sleep (important in recovery) in NREM significantly increased in valerian group -Positive effects on mild psychological insomnia	no mechanism of action presented	17
**V. Officinalis**	Sedonium: Li 156 extract: an alcoholic extract, by use of 70% ethanol of dry root of European V. officinalis. Each tab: 300mg	Randomized, Double-blind, Placebo-controlled	3	Valerian 300 mg, 600 mg, placebo EEG recorded at 23:00	16 patients (50-64y) have a mild sleep complaint	3weeks. 6days washout between 2doses	No significant differences between 300 mg and 600 mg valerian and placebo, in sleep EEG, mood, psychometric performance	no mechanism of action presented	18
**Valeriana edulis** **(Valeriana Mexicana)**	Rhizome and root/ Hydroalcoholic extract contains valepotriate acid dose not contain valerenic acid	Randomised, double-blind, the crossover study	3	Which patients received both treatments: 150mg dried extract of V. edulis and 150 mg V. officinalis as control, 3times a day (450mg)	20 patient (29-55y) with insomnia accordance with DSM-III-R	4nights	-↑REM sleep (more by Officinalis) -↑delta activity -↓stage 1,2 in NREM sleep - ↓sleep latency, awakening episode, morning sleepiness(more by Officinalis)	no mechanism of action presented	19

**Table 2:** Animal Studies for Insomnia

**Scientific name (common name)**	**Part** **(extract)**	**Model**	**Intervention**	**Duration**	**Animal type**	**Result**	**Mechanism**	**Reference**
**Piper methysticum**	Root (kava-kava is an extract of the roots of the Piper methysticum) -96% ethanolic extractvs	By placing rats on a grid that was suspended over water	kava-kava extract at a dose of 30,10,300 mg/kg or flunitrazepam at doses of 1,3 and 10 mg/kg both suspended in 0.5% carboxymethyl cellulose solution and administered orally at 9:00 -Antagonism test: flumazenil in 10% dimethyl sulfoxide injected before drug test -control: sleep-deprived rats	7 day intervals	Male rats	-↑delta activity in NREM sleep (against flunitrazepam) -No effects on the total waking and NREM sleep time - Significantly ↓sleep latency	The extract is not mediated through the benzodiazepine receptors (because the effect was not antagonized with flumazenil)	20
**Matricaria chamomilla/** **Passiflora incarnate**	Flower parts of chamomile / aerial parts of passiflora	placing rats on a grid in a cage filled water to 1cm below the grid surface	Received 3,10,300 mg/kg chamomile and300,1000,3000 or mg/kg passiflora Suspended in 0.5% carboxymethyl cellulose orally -Antagonism test: flumazenil in 10% dimethyl sulfoxide injected before drug test -control: sleep-deprived rats	7 day intervals	Male rats	- ↓sleep latency by chamomile that antagonizes by 3mg/kg flumazenil -No effects on NREM and REM sleep, Delta activity, wakefulness time	Chamomile has BZD-like hypnotic activity (the effect was antagonized with flumazenil)	21
**Valeriana officinalis**	Root ethanolic extract	Placing rats on a grid suspended over water	1000,3000mg/kg dissolved and administered then EMG and EEG measured 6h after administration. -control: sleep-deprived rats	7 day intervals	Male rats	-↓sleep latency (dose dependent) -↑delta activity in NREM sleep -No effects on NREM, REM, Wakefulness	no mechanism of action presented	22
**Ganoderma lucidum**	Fruiting bodies		-40 and 80 mg/kg -TNFα 12.5, 25 ng/rat i.c.v alone and coadministration by G. lucidum (40 mg) -TNFα antibody (2.5 g/rat, i.c.v.) were injected 20 min prior to the last G. lucidum (80mg) application.	3 days	Male rats	-↓sleep latency -↑ total sleep time and NREM -No effects on SWS and REM -↑TNFα in serum, the hypothalamus, and dorsal raphe concomitantly	may be related, at least partially, to the TNFα pathway Like modulation of cytokines such as TNFα	23
**Hypericum perforation** **(St. Johns Wort)**	Ethanolic extract	72h Sleep deprived by placing them on the grid suspended over water	- (200mg and 400mg/kg PO) - Imipramine (10mg/kg, i.p.) -Imipramine 10 +200 st.johns wort -control: sleep-deprived rats - starting 2 days before sleep deprivation	5 days	Male mice	-↑body weight, locomotor activity, antianxiety effect -↓oxidation damages (co-administration with imipramine: greater improvement	Inhibit serotonin dopamine NE reuptake (similar to SSRIs and TCAs), Antioxidative effects by polyphenolic acids and flavonoids -imipramine has antianxiety effects as well as st.john's wort	24
**Coriandrum** **sativum**	Aerial parts /hydro-alcoholic extract three fractions: water (WF), ethyl acetate (EAF) and N-butanol (NBF)		- Pentobarbital 30 minutes after injection of saline, diazepam, or C. Sati vum extract (HAE); vs. saline - Vehicles(saline), or 50 mg/kg of HAE fractions: (WF), (EAF) and (NBF) before injection of pentobarbital;		Mice	-HAE and EAF, NBF(more) ↑sleep duration, -NBF↓sleep latency - NBF showed the highest hypnotic activity -No neurotoxic effect	no mechanism of action presented	25
**Panax ginseng** **(Korea Red Ginseng)**	Water extract		200 mg/kg orally by dissolving in distilled water once per day	7 days of post-surgical recovery+9 days trial	Male rats	↑total sleep and NREM sleep ↓wakefulness, sleep-wake cycles -↑α-wave activity during NREM and REM -↓ the expression of α- and β-subunits of GABA receptors	Modulates sleep via the GABAAergic systems	26
**Panax ginseng** **(Korea Red Ginseng)**	Six-year-old red ginseng roots/ ethanol extract		Oral administration of 10,50,100mg/kg was performed 10 min before EEG recording	7 days	Male rats	-10 mg/kg ↑α-wave activity, ↓δ-wave activity in REM and NREM sleep,↑ significantly NREM and total sleep -50 mg/kg like 10 mg but without any effect on REM -100 mg/kg no effect on EEG waves just ↑ total sleep -Lower doses of RGE are more effective at modulating sleep and specifically affected NREM sleep.	no mechanism of action presented	27
**Valerian preparation (BIM)**	-Root of V. officinalis + Rhodiola rosea (golden rose) + L-Theanine (γ-glutamylethylamide) -Ethanolic extract	A grid floor was filled with water	100,300,1000 mg/kg of BIM :(400mg valerian+100mg golden root +50mg L-theanine) dissolved and administration at 9:00 or - valerian(30,1000 mg/kg) alone	7-day intervals	Male rats	-↓sleep latency (sig in 1000mg of each one and more effect for valerian than BIM) -no effect on NREM, REM,wakefulness and delta activity in NREM sleep for both	↑GABA level for valerian	28
**Stephonia**	L-stepholidine, an active ingredient		I.p, bolus Injected doses of 20, 40 or 80 mg/kg stepholidine -vehicle (20ml/kg) as control -diazepam 6mg/kg	2 days (Experimental and baseline day)	Male mice	↓sleep latency to NREM -↑ the amount and duration of NREM sleep by 40,80mg/kg -↓wakefulness -40 and 80 mg/kg ↑number of state transitions from wakefulness to NREM sleep and from NREM sleep to wakefulness.	Mixed partial dopamine D1 receptor agonist/full D2 antagonist properties - promoted NREM sleep, blockade of D2R plays a major role in the hypnotic action of SPD - Activation of sleep-active neurons in the VLPO	29

**Table 3:** Combination Therapy for Insomnia

**Scientific name (common name)**	**Part (extract)**	**Study design**	**Jadud** **score**	**Intervention**	**Patients**	**Duration**	**Result**	**Mechanism**	**Reference**
**Valeriana officinalis + Humulus lupulus**	-Dormeasan®: 460 mg of V. officinalis+ 460 mg H. lupulus -1: 12 dissolved in 61% ethanol	Double-Blind, Randomized, Placebo-Controlled	3	2 ml dissolved in 50 ml of honey flavored water and administrated orally 15 min before EEG(n=20) -placebo(n=22)	44 patients (30-70y) reporting on having a poor sleep for 2 weeks without neurological complications and any organic disease	2nights(reference night and medication night).	-Led to deeper sleep. -↑sleep quantity in the treatment group	no mechanism of action presented	30
**V. officinalis + H. lupulus**	Ze 91019: 500 mg valerian extract siccum + 120 mg hops extract siccum _45% methanol m/m with a drug extract ratio of 5.3:1 (valerian) and 6.6:1 (hops)	Randomized, Double-Blind, Placebo- Controlled	2	-Valerian Ze 911 (n=10) - Valerian/hops Ze 91019 (n=10) -placebo (n=10)	30 patients (≥18 years) suffering from non-organic sleep disorders (ICD 10, F 51.0–51.2)	4weeks	-No significant differences across groups in NREM -↑significantly sleep latency for Ze91019 -single valerian is not superior to placebo in sleep latency (=plausibility for adding hops extract to the valerian Extract)	decreasing excitatory neurotransmission in the central nervous system	31
**Hypericum perforatun** **(St. Johns Wort)** **+ V. officinalis** **+ Passiflora** **Incarnata** **(passionflower)**	Neurapas balance: 60mg St. John’s wort+28mg valerian+ 35mg passion flower	Double-blind randomized cross-over study		3 tablets twice a day	20 healthy subjects	3days	-↓ wakefulness, REM latency, NREM sleep in the first sleep cycle -↑NREM- sleep in the second cycle -↑subjective mood and subjective sleep evaluation -no difference in cognitive performance	no mechanism of action presented	32
**Valeriana officinali + Humuluslupulus**	Dormeasan®:460 mg of fresh V. officinalis; radix (root) rec. Tinct. 1:10 and H. lupulus, strobulus (fruit) rec. tinct. 1:12 dissolved in 61% ethanol	Double-blind, randomized, placebo-controlled	3	-2ml of the formulation was dissolved in 50 mL of honey-flavored water and then administered orally(n=20 -placebo (n=22)	42 healthy (Mean age of female 50.2 and males 48.2) having poor sleep within the previous 2weeks period	2 consecutive nights (reference nd medication night)	-↑sleep time and deeper sleep time Significantly	May: 1.inhibit central catabolism of GABA 2.bind directly to GABA-A receptors and stimulate the release and reuptake of GABA	33
**Piper methysticum (kava) / Valeriana officinali**		crossover trial, not double-blind	0	First treated for 6 weeks with kava 120 mg daily(n=24) This was followed by 2 weeks off treatment and then, 5 having dropped out, 19 received valerian 600mg daily for another 6 weeks (n=19)	24 patients suffering from stress-induced insomnia ( 23-65y)	6 weeks for each drug with washout period of 2weeks between	-↓Total stress severity by both -valerian: no sig effect on inducing sleep but good effect on quality -stress problems and the severity of the resulting insomnia were rapidly relieved by kava -improvements achieved with kava were then maintained by valerian	no mechanism of action presented	34
